# Homeostasis and Compensatory Homeostasis: Bridging Western Medicine and Traditional Chinese Medicine

**DOI:** 10.2174/157340311795677671

**Published:** 2011-02

**Authors:** Xiu-Juan Fan, Hao Yu, Jun Ren

**Affiliations:** 1 Center for Cardiovascular Research and Alternative Medicine, University of Wyoming College of Health Sciences, Laramie, WY 82071, USA; 2 China Nepstar Chain Drugstore Ltd., Hangzhou 310003 Zhejiang, China; 3 Ren Zhi Tang Clinic of TCM, Shiyan 442000 Hubei, China

**Keywords:** Homeostasis, compensatory homeostasis, western medicine, traditional chinese medicine.

## Abstract

Compensation is a self-protective mechanism in diseases, which may lead to a unique form of homeostasis deviates from that in physiological conditions. The kind of compensatory homeostasis can be embodied as various degrees accompanying disease progression (denoted as compensatory degree). Compensatory homeostasis provides a window for the transition from disease to healthy state. The causes of compensatory homeostasis themselves may be identified as targets for effective measures to eliminate compensation. Compensatory homeostasis embodies significantly mostly in the developing process of chronic diseases, which may help to explain in theory why intensive therapeutic strategies led to unexpected outcome in clinical practice. In addition, a large body of clinical evidence has valued traditional Chinese medicine (TCM), which is based on shifting compensatory homeostasis to the overall human body homeostasis, complementary to Western medicine in the management of chronic disease. In this review, we will briefly summarize the concept of compensation and attempt to bridge Western and traditional Chinese medicine through homeostasis and compensatory homeostasis based on an ample of evidence obtained from both disciplines

## BACKGROUND

Compensation is a self-protective process to maintain the whole physiological function in the circle of life. Many phenomena including cardiac hypertrophy in heart failure, vasospasm or microvascular spasm and ischemic hypoxia in shock, are all excellent examples of the compensation machinery in human body. Metabolic homeostasis is maintained through compensation during the pathological phase. For example, glucose transporters are located at the membrane of cardiomyocytes with GLUT4 being the predominant type in hearts and function as a key regulator of glucose uptake, energy metabolism and cardiac function in ischemic myocardium [1]. Interestingly, both mRNA and protein expression of GLUT4 are upregulated under myocardial ischemia, associated with a translocation of GLUT4 from cytosol to plasma membrane [2]. These changes allow facilitated glucose uptake in the ischemic myocardium to maintain energy supply in the ischemic region. Upregulated GLUT4 expression and facilitated membrane translocation in response to ischemic insult are compensatory mechanisms to maintain homeostasis in myocardial ischemia through preserving the cardiac pump function.

## COMPENSATORY HOMEOSTASIS AND COMPENSATORY DEGREE

Although compensation helps to maintain homeostasis in a living organism, the homeostasis in pathological conditions is drastically different from that in healthy conditions.Homeostasis under diseases is a type of compensatory “homeostasis” deviated from the healthy one. We may define the degree of deviation as “compensatory degree”. Compensatory homeostasis exists between healthy conditions and contabescence of life. A low degree of compensation means a small degree of compensatory homeostasis deviated from the healthy one, which might be one or a few minor or regional feedback loops damaged and accompanied by corresponding compensatory pathway(s) [or compensatory loop(s)]. This type of illness should be easier to be treated with a better prognosis. However, in the case of a large degree of compensatory homeostasis deviated from the healthy one, or large compensatory degree, where many and/or crucial feedback loops may be impaired and then followed by the initiated multiple corresponding compensatory pathways or loops, the problem or disease may be more difficult to cure with an unfavorable prognosis. During early stages of disease progression, compensation is often embodied with intrinsic functional compensation. Over time, however, structural changes may develop in the affected tissues or organs manifested as “structural compensation”, such as left ventricular hypertrophy in hypertension, progression of atherosclerotic plaque in late stage hypertension and/or dyslipidemia [3,4]. During the course of disease progression, compensatory homeostasis may transition gradually and smoothly without any obvious sign. Any given significant deterioration may be the result of a broken compensatory homeostasis followed by establishment of a new compensatory homeostasis with a larger compensatory degree. For example, hyperinsulinemia may be triggered by insulin resistance *en route* to full-blown type 2 diabetes, leading to the development of poorly controlled ketoacidosis [5]. All these different stages in disease progression may reflect a newly broken compensatory homeostasis followed by a subsequent larger compensatory homeostasis. Compensatory homeostasis embodies homeostasis in the human body as the old Chinese proverb “Boat goes up as river rises”, especially in the course of progression of systematic diseases including hypertension, diabetes and dyslipidemia.

Compensatory homeostasis and the compensatory degree theory have been described as we mentioned above. Nonetheless, one question remains to be answered is that if they really exist in a living organism. In clinical practice, an abrupt drop in either blood pressure or blood glucose (drug-induced) often leads to hypotensive or hypoglycemic coma. Dramatic lipid-lowering readily induces geriatric psychological and emotional shift or cardiovascular events. Meanwhile, a “plateau phase” in either blood pressure or body weight may occur during drug treatment for hypertension or obesity probably due to the onset of regulatory compensation [6]. The human body is in a compensatory state when administration of an antihypertensive, lipid-lowering or oral hypoglycemic drug or injection of exogenous insulin occurs. However, the upregulated compensatory pathways especially accompanied with structural changes cannot keep pace with the dramatic decrease triggered by overdose or intensive drug usage to maintain the dynamic compensatory homeostasis, representing a state of “broken compensatory homeostasis”. It is a similar scenario to lower the height of boat merely without considering the elevated water level. There should be little overt difference between intensive and proper/standard therapy if there was not a homeostasis or rather compensatory homeostasis. Sudden interruption of the compensatory homeostasis especially with structural change in tissues or organs is expected to elicit serious consequences. This may explain why there was a higher mortality rate with more aggressive compared with the standard glucose-reducing treatment [7-9]. Similarly, other evidence also consolidated the existence of compensatory degree [10-16]. In a study where human HeLa cells were exposed to the antiretroviral nucleoside reverse transcriptase inhibitor (NRTI) Zidovudine1 (AZT) for up to 77-passages (p77), abnormal mitochondrial proliferation was seen at p5 and aberrant morphology developed at passages after p36 although the increased mtDNA quantity was present at early stages. Approximately 65% mtDNA quantity was depleted at p71 in conjunction with the elevated mitochondrial membrane potential at early stages and essentially absent at p71 [16]. The data indicated different degrees of a compensatory response at the earlier passages and a profound mitochondrial morphological damage and loss of mitochondrial membrane potential at the late stage, suggesting the existence of “compensatory degree”. Another representative example of compensatory homeostasis and compensatory degree in patients is the increased endothelial progenitor cells during early stage of heart failure and then decreased number of endothelial progenitor cells in advanced heart failure [17,18]. Further experimental and clinical evidence is warranted to consolidate this theory.

## HOMEOSTASIS AND SELF-HARMANIZED YIN-YANG

Compensatory homeostasis described here represents conditions in pathological or pathophysiological state. In the case of physiological conditions, that is homeostasis, a term coined by American physiologist Walter B. Cannon to describe the constancy of an internal environment, a concept first introduced by French physiologist Claude Bernard [19]. It is nowadays widely employed to represent dynamic equilibrium in organisms include humans, for instance, homeostasis of glucose, cholesterol, energy, etc, which is regulated by feedback primarily. Homeostasis, in fact, the concept has been elucidated in depth in Traditional Chinese Medicine (TCM) in another term, self-harmonized Yin-Yang. According to the “Treatise on Cold Damage Diseases” (Shang Han Lun) by Zhongjing Zhang, a notable classic of TCM three century A.D., if patients with cold damage diseases are induced to sweat, or vomit, or catharsis, and in the event of loss of blood or body fluid, the patients whose yin-yang may be self-harmonized will be *en route* to the self-healing of the diseases. The self-harmonized Yin-Yang means conditions that human body is regulated by feedback. From the earlier classic of TCM – “Inner Canon of Huangdi” (Huangdi Neijing), an essential factor for the Yin-Yang harmony and the overall well-being is a strong enough Yang to guard outside. It seems like spring without accompanied autumn, winter with-out accompanied summer when they are not harmonized, therefore, harmonizing them is the quality of monarches.

**Scheme (1):**
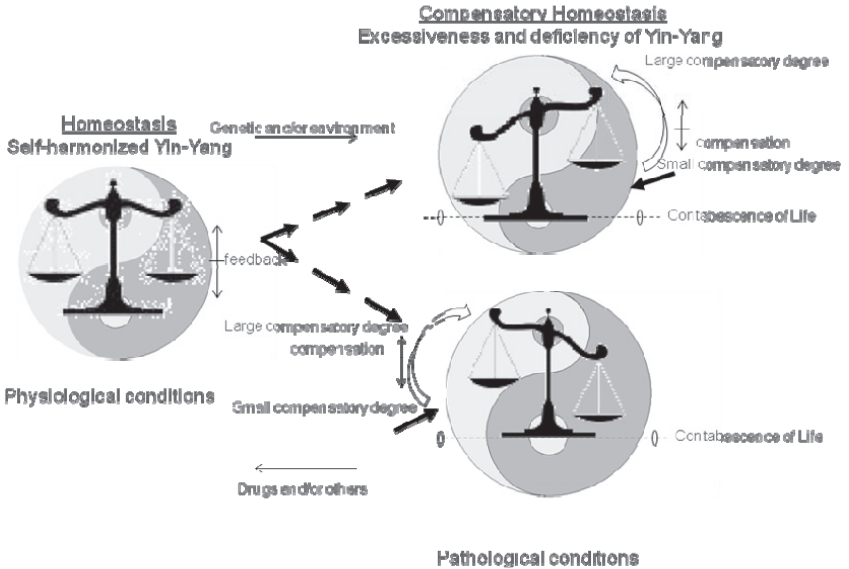
Homeostasis/Self-harmonized Yin-Yang and Compensatory homeostasis/Excessiveness and deficiency of Yin-Yang.

The Chinese characters Yin-Yang represent the two opposite inter-dependent characteristics or things. They have been summarized as the principle of the universe, the origin of changes and life and the intrinsic law of all things in the universe. Yang transforms to Qi, and Yin condensates to substantial material in the human body according to “Inner Canon of Huangdi”. For example, “Yin” resembles cold, stillness, darkness, passivity, inwardness and downwardness, On the other hand, “Yang” represents heat, movement, brightness, activity, outwardness and supwardness [20]. A schematic diagram depicting such relationship is illustrated in Scheme **1**. Similarly, such paired Yin-Yang at different levels is common in human body. For instance, the double strands of DNA double helix; Kinases (yang) and counterbalanced phosphatases(yin) of cellular proteins [21, 22]. Additional examples of the paired Yin-Yang includes the regulation of eIF2α phosphorylation in islet β cells involving eIF2α phosphorylation and global ER translation [23], the pro-oxidants and anti-oxidants in lipoproteins (such as cholesteryl ester versus vitamin E) [10], the “*Yang-regulators”* Ang II, endothelin, and aldosterone and reciprocal counterbalanced “*Yin-regulators”* bradykinin, prostaglandins, nitric oxide, atrial natriuretic peptide and glucocorticoids [24].

## COMPENSATORY HOMEOSTASIS AND EXCESSIVENESS AND DEFICIENCY OF YIN-YANG

In pathological or pathophysiological conditions, that is compensatory homeostasis, which is regulated by compensation primarily at least in imbalanced parts although feedback regulation is still exist in physiological regions of human body. Compensatory homeostasis will be shifted to homeostasis by eliminating the cause(s) of compensation instead of targeting the compensatory pathway(s), diseases will be cured. Factually, both the concept and theory of “compensatory homeostasis” and correction of compensatory homeostasis to homeostasis also have been elucidated in depth in TCM in its own terms, the excessiveness and deficiency of Yin-Yang. The excessiveness of Yin denotes the illness of Yang, while the excessiveness of Yang indicates the damage of Yin from the ‘Inner Canon of Huangdi”. It is believed that the Yin-Yang disharmony may be the basic cause of pathogenesis of diseases in TCM [20], which caused excessiveness and deficiency of Yin-Yang. Accordingly, harmonize the Yin and Yang, is deemed the general principle of treatment for diseases in TCM [20]. The excessiveness and deficiency of Yin-Yang fits nicely with the compensatory homeostasis. (A schematic diagram depieting such relationship is illustrated in Scheme **1**). The treatment philosophy of TCM aims at shifting compensatory homeostasis to homeostasis *via* a global adjustment of the human body, similar to lower the height of boat by decreasing water level synchronously. In detail, “the Yin-Yang is examined to discern the weakness and strength, and then the Yin is treated for the diseases of Yang category, and the Yang is interfered for the illness of Yin category from “Inner Canon of Huangdi”. For the excellent acupuncturists, a common practice is being used to induce Yang from Yin, to induce Yin from Yang, to treat the left *via* the right, and to treat the right *via* the left. From the book “Required Readings for Medical Professionals” by Zhongzi Li nearly 400 years ago, it is recommended to avoid targeting the sputum, blood, sweat, heat, and panting while those are merely phenomena.

## SUMMARY

In summary, as shown by both the classical Diagram of Tai Chi Yin-Yang and balance in Scheme **1**, the human body is the converging point between the Traditional Chinese Medicine and Western medicine. Concepts such as homeostasis/self-harmonized Yin-Yang, compensatory homeostasis/excessiveness and deficiency of Yin-Yang may build a bridge between the two disciplines. It is essential to communicate between the two to make them complement each other, including the cutting edge research findings from Western medicine, therapeutic theory and evidence-based practice from the Traditional Chinese Medicine, in order to promote the development of medicine greatly. Provided that compensatory homeostasis and compensatory degree do exist universally in diseases, the gradually modest instead of intensive therapeutic strategies are especially valued during the treatment courses of the chronic systematic diseases exemplified by hypertension, diabetes and dyslipidemia.
