# Erratum

**DOI:** 10.2174/157340312803217210

**Published:** 2012-08

In the article by Fan *et* *al*, “Homeostasis and Compensatory Homeostasis: Bridging Western Medicine and Traditional Chinese Medicine” which was published in the February 2011 issue of the journal (*Curr Cardiol Rev* 2011; 7:43-46 43-46), an institution was wrongly listed for the author affiliations.

The author affiliations should appear as follows:

Xiu-Juan Fan^1,2^, Hao Yu^3^ and Jun Ren^2^

^1^China Nepstar Chain Drugstore Ltd., Hangzhou 310003 Zhejiang, China; ^2^Center for Cardiovascular Research and Alternative Medicine, University of Wyoming College of Health Sciences, Laramie, WY 82071, USA; ^3^Ren Zhi Tang Clinic of TCM, Shiyan 442000 Hubei, China

Address correspondence to this author at China Nepstar Chain Drugstore Ltd., Hangzhou 310003 Zhejiang, China; Tel: +86-0571-86872908; Fax: +86-0571-86872929; E-mail: janetscience@gmail.com and this author at the Center for Cardiovascular Research and Alternative Medicine, University of Wyoming College of Health Sciences, Laramie, WY 82071, USA; Tel: +1-307-766-6131; Fax: +1-307-766-2953; E-mail: jren@uwyo.edu

There were also several wrong translations about traditional Chinese medicine.

Those translations should appear as follows:

“Treatise on Exogenous Febrile Diseases” (Shang Han Lun) by Zhongjing Zhang, a notable classic of TCM three century A.D., if patients are induced to sweat, or vomit, or catharsis, …

Excess and deficiency of Yin-Yang, as shown in the Scheme **1**


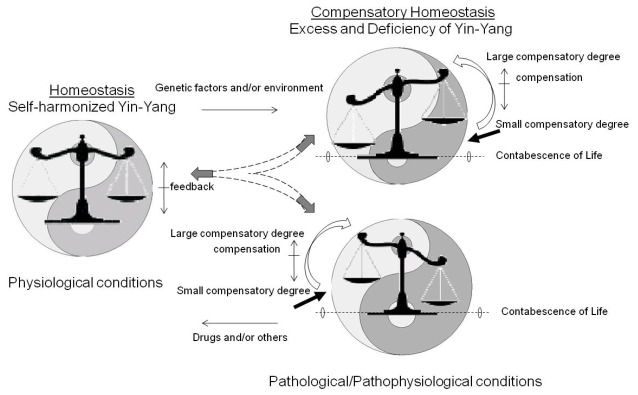


The online version of the article has been corrected. The authors apologize for the errors.

